# Supplementary material for: Expanding canonical cortical cell type markers in the era of single-cell transcriptomics
Source: Sci Rep. 2026 May 21;16:24601. doi: 10.1038/s41598-026-51501-2 (PMC13454430; doi:10.1038/s41598-026-51501-2)
Supplement: Supplementary file 1 [file 41598_2026_51501_MOESM8_ESM.pdf]

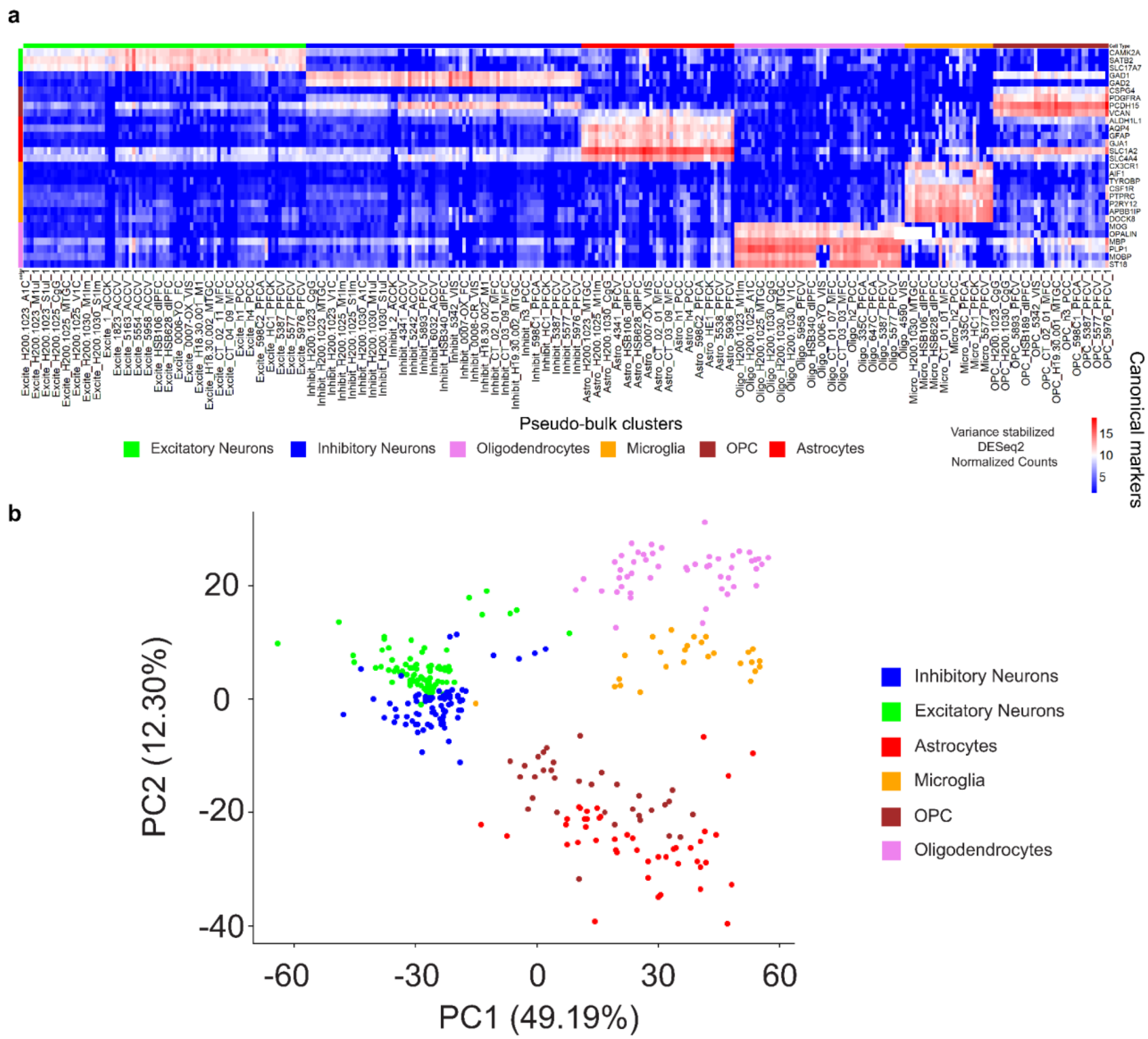

Supplementary Figure S1: a) variance stabilized, normalized global expression matrix in heatmap form for plotted using all 319 pseudo-bulk clusters demonstrate varying levels of marker expression and potential background gene expression in some canonical markers in other cell types. variance stabilized, normalized global expression matrix in heatmap form for plotted using all 319 pseudo-bulk clusters, with canonical markers on the y-axis. b) PCA plot of pseudo-bulk aggregated cell type-sample clusters show clear clustering in the first two PCs (calculated using DESeq2 normalized data, followed by VST transform and selecting the top 2000 variable genes), with both neuronal types in proximity, glial cell types close to each other and the microglial cluster

apart from the 5 other cell types.

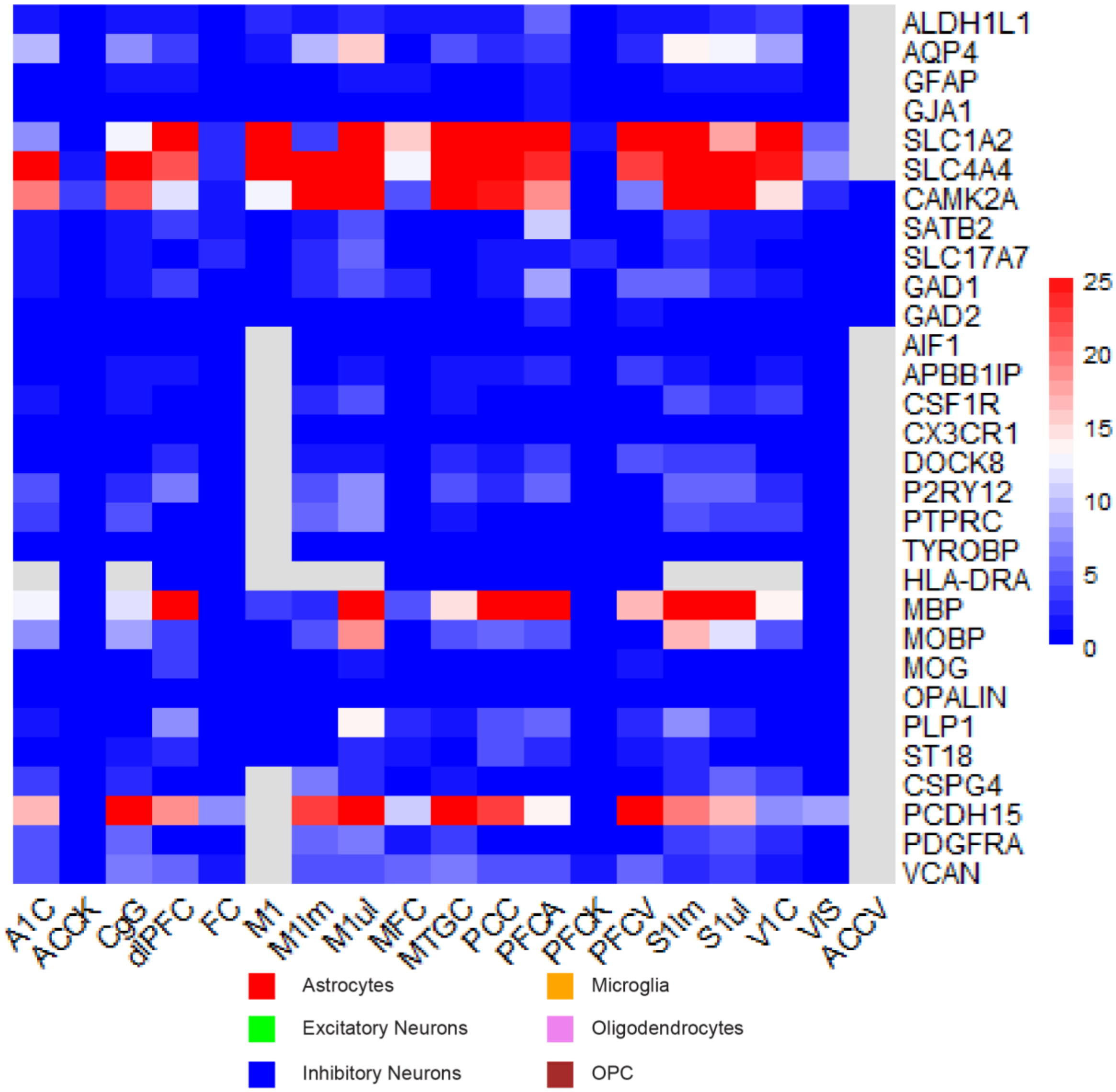

Supplementary Figure S2: Some canonical cell type markers have significant background signals that vary across regions.

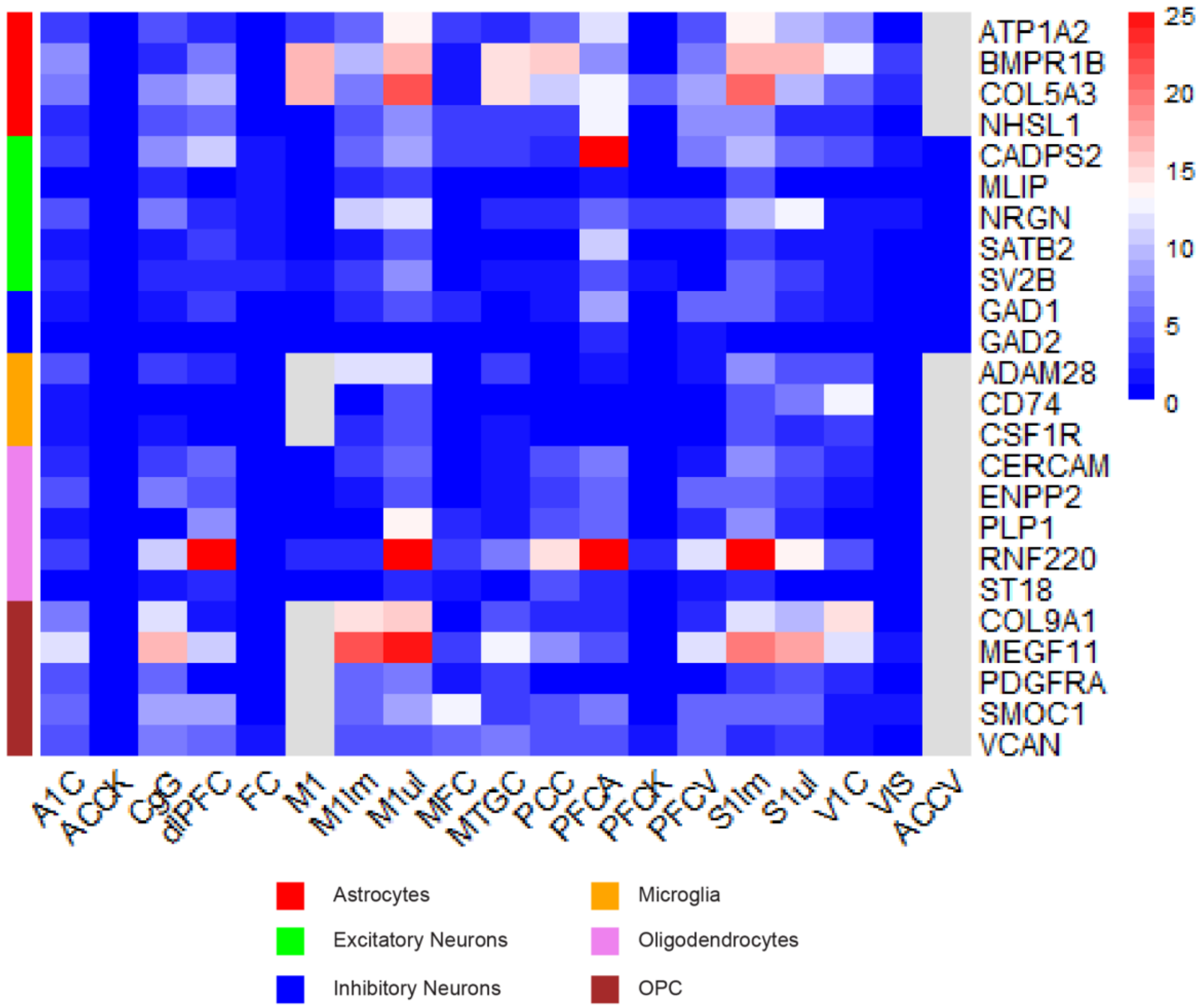

Supplementary Figure S3: Discovered calculated markers have lower or similar background signals as canonical cell type markers.

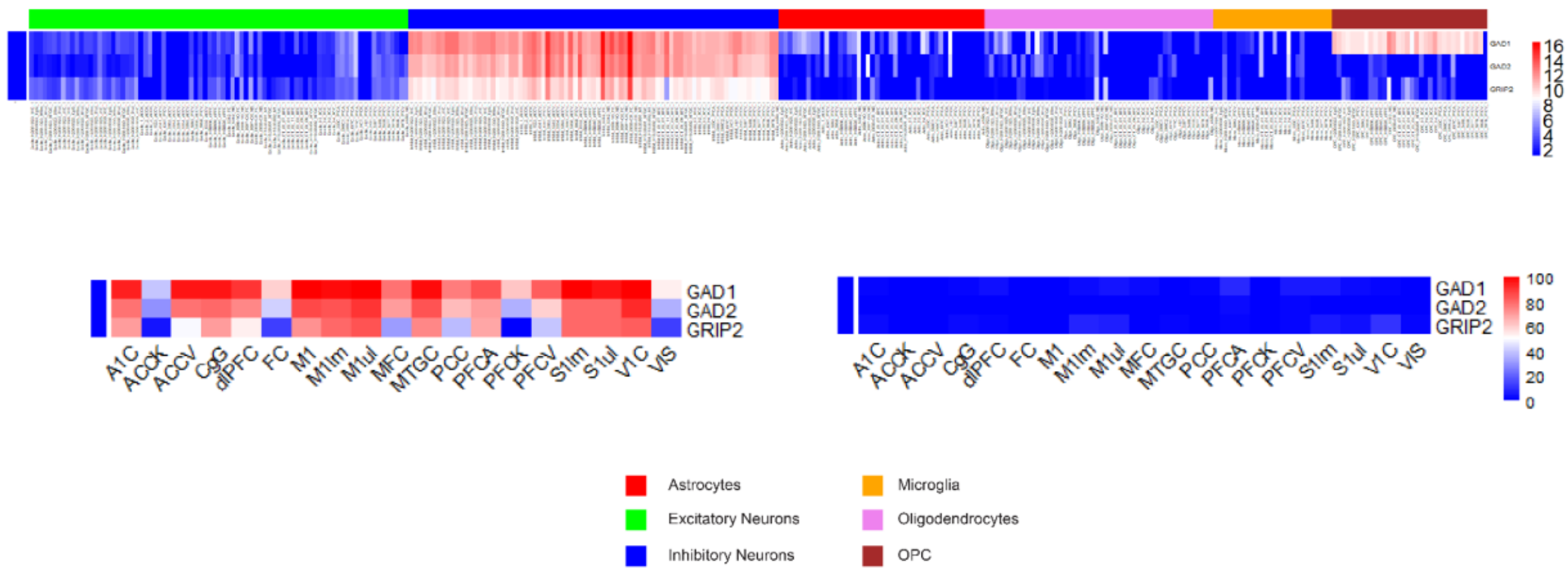

Supplementary Figure S4: *GRIP2* is a potential pan-inhibitory neuronal marker at par with *GAD2*, but has high variability in fidelity (lower left), but matches the background profile (lower right) of *GAD1* and *GAD2*.

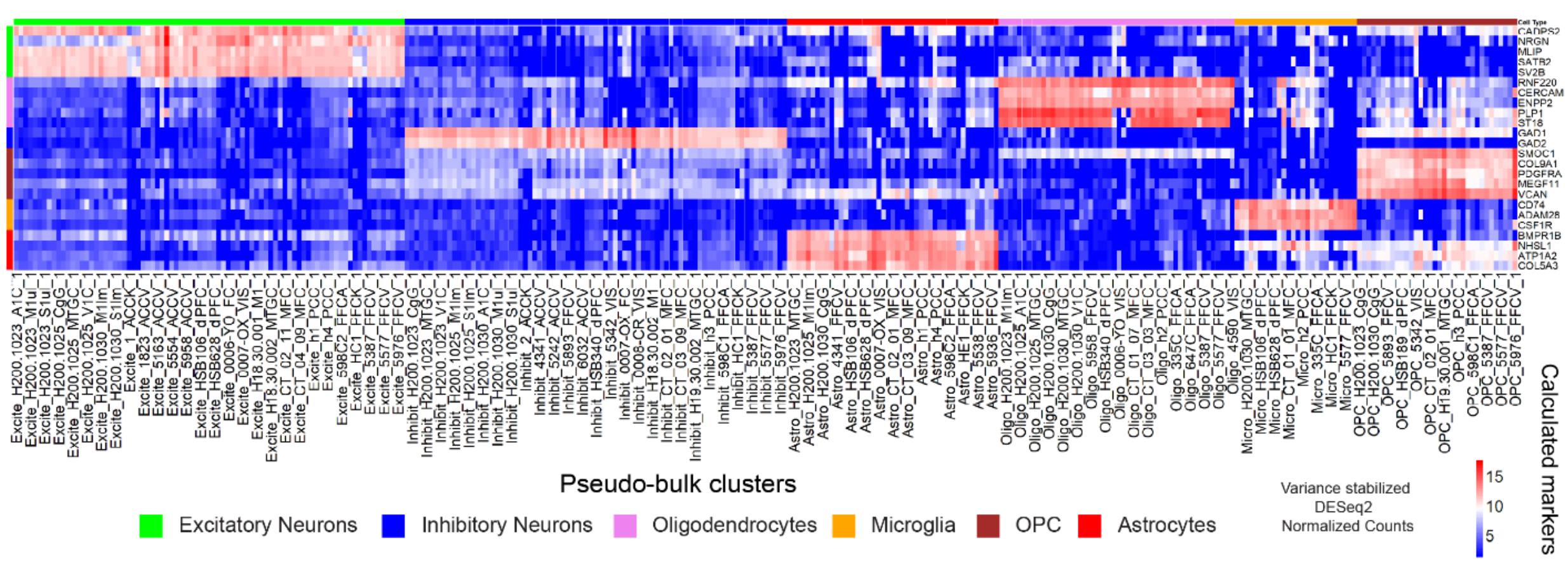

Supplementary Figure S5: Calculated markers clustered pseudo-bulk clusters of cell types with high ARI and average Silhouette score.

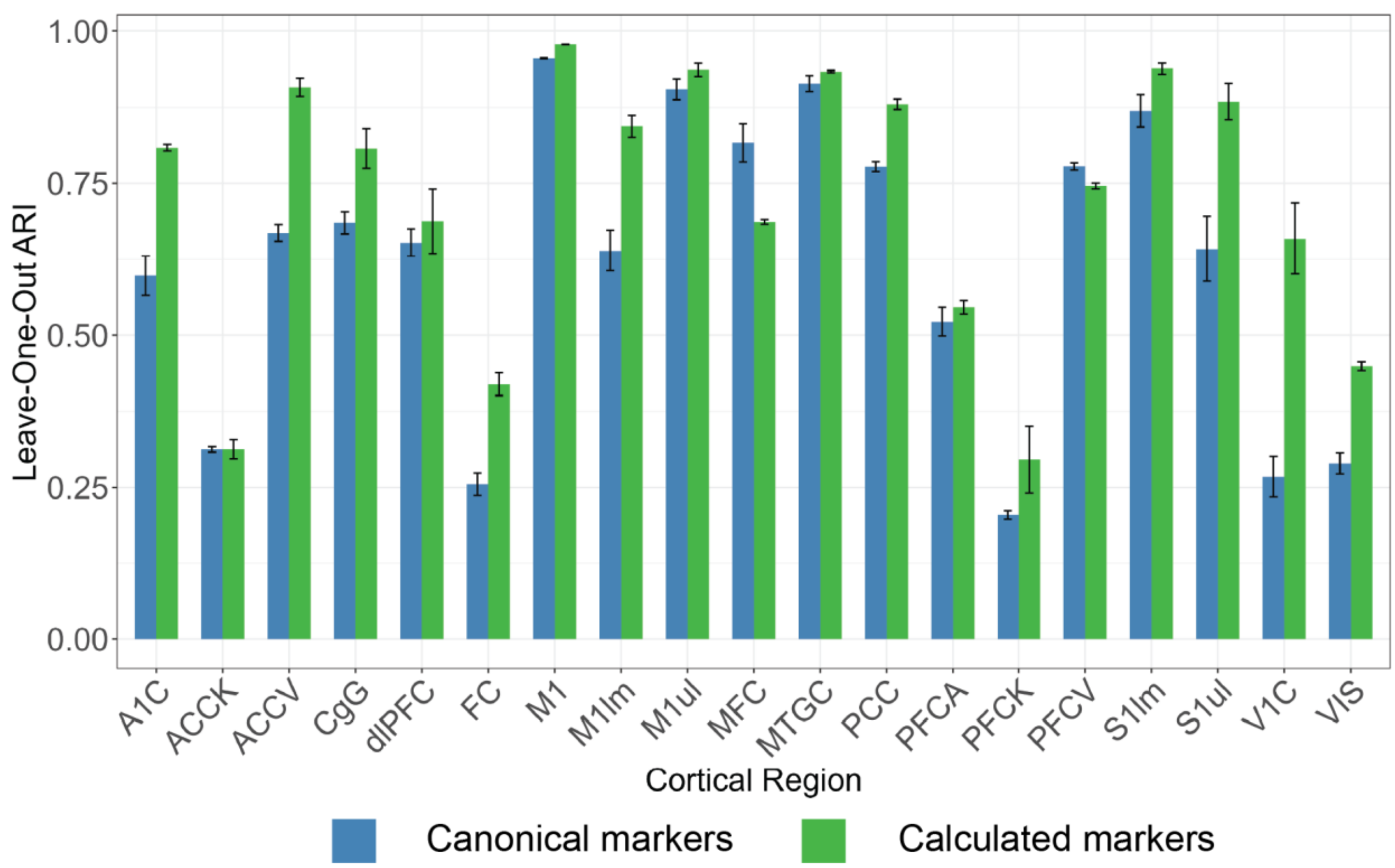

Supplementary Figure S6: Calculated markers perform better or at par with canonical markers with higher ARI scores in 16/19 regions compared to canonical cell type markers.

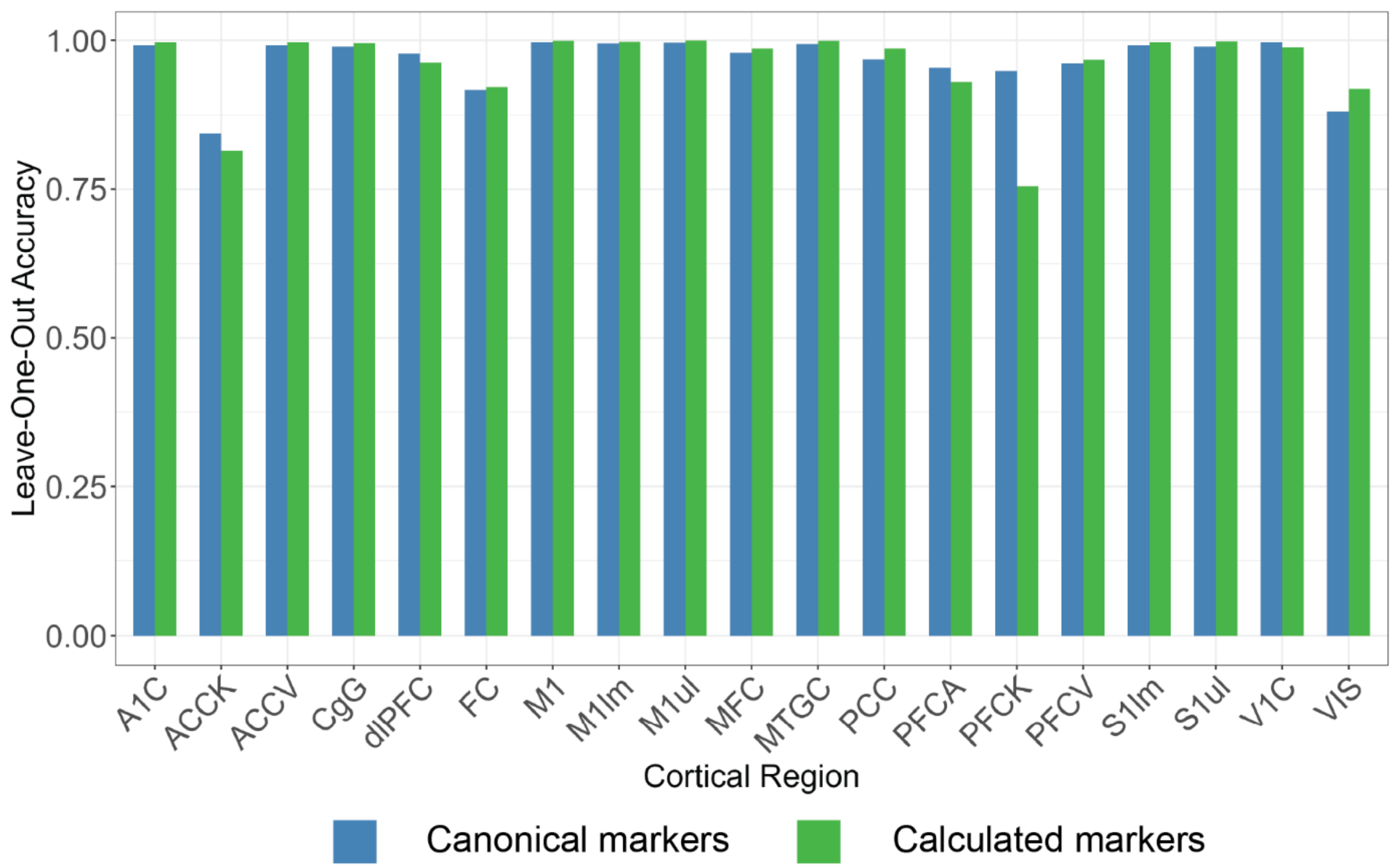

Supplementary Figure S7: Calculated markers outperform canonical markers with higher accuracy scores in 14/19 regions compared to the canonical cell type markers

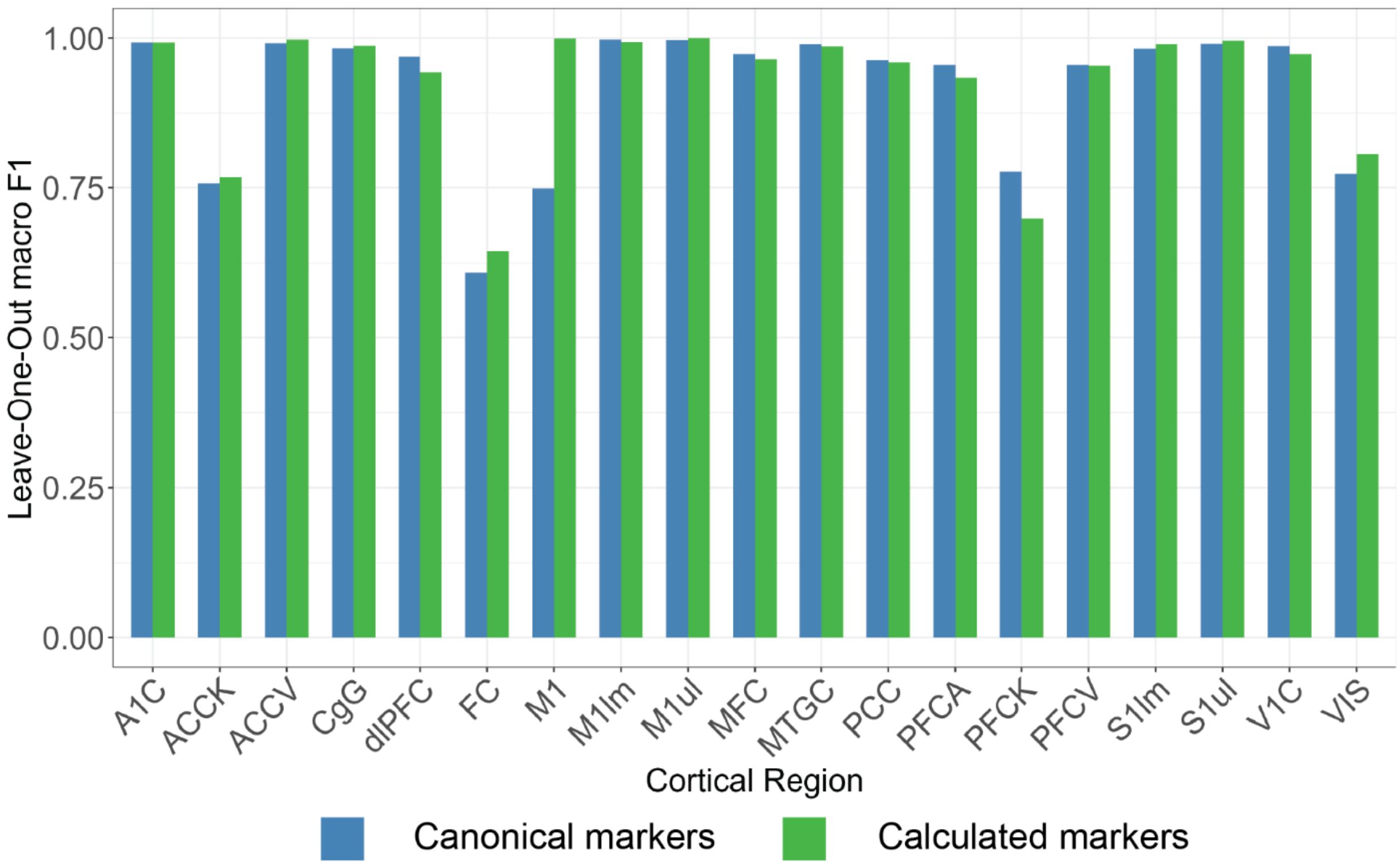

Supplementary Figure S8: Calculated markers outperform canonical markers with higher average macro F1 scores in 9/19 regions compared to the canonical cell type markers

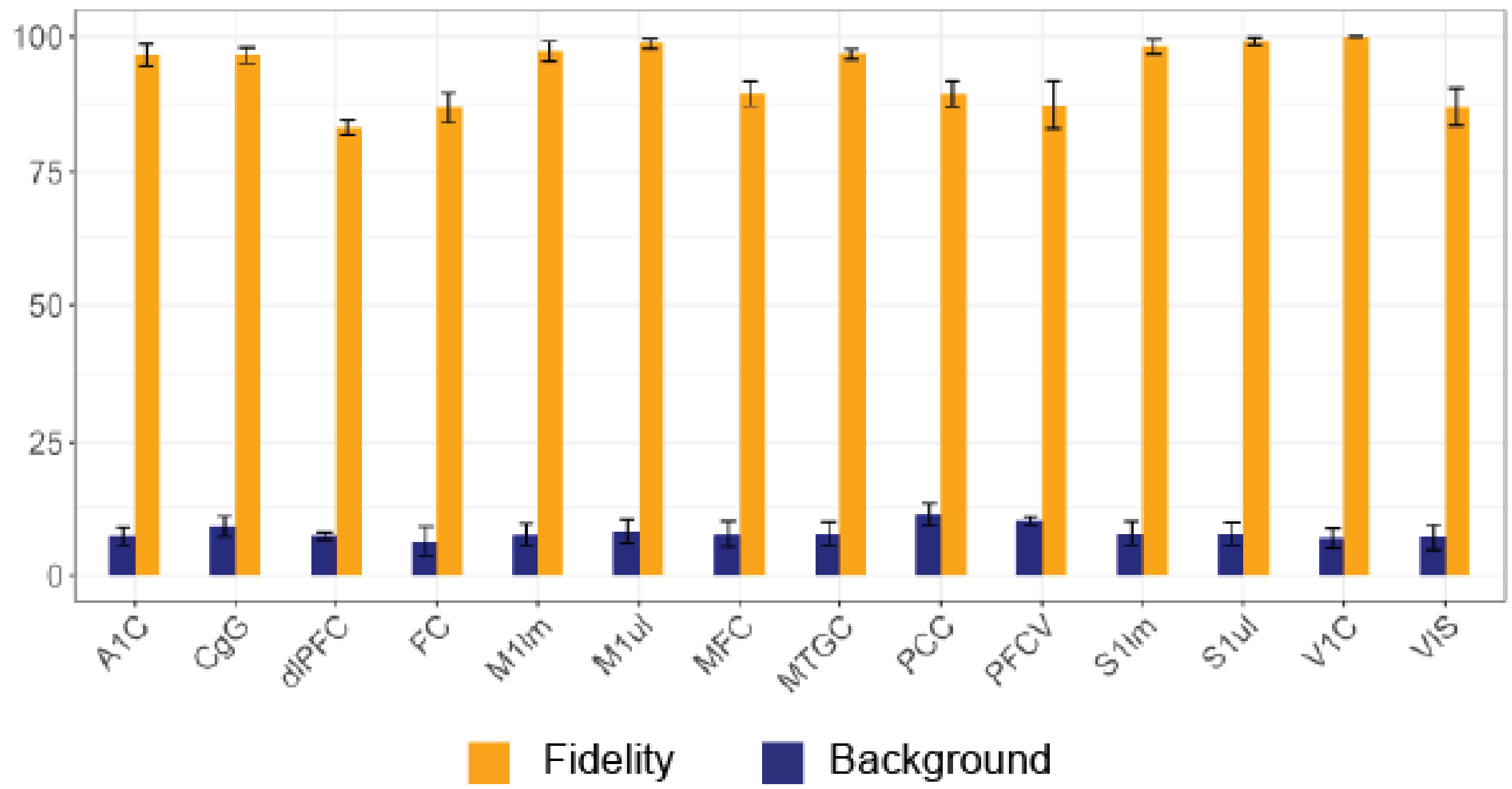

Supplementary Figure S9: High fidelity and low background levels of astrocyte subtype markers calculated using our method across all studies with subtype resolution.

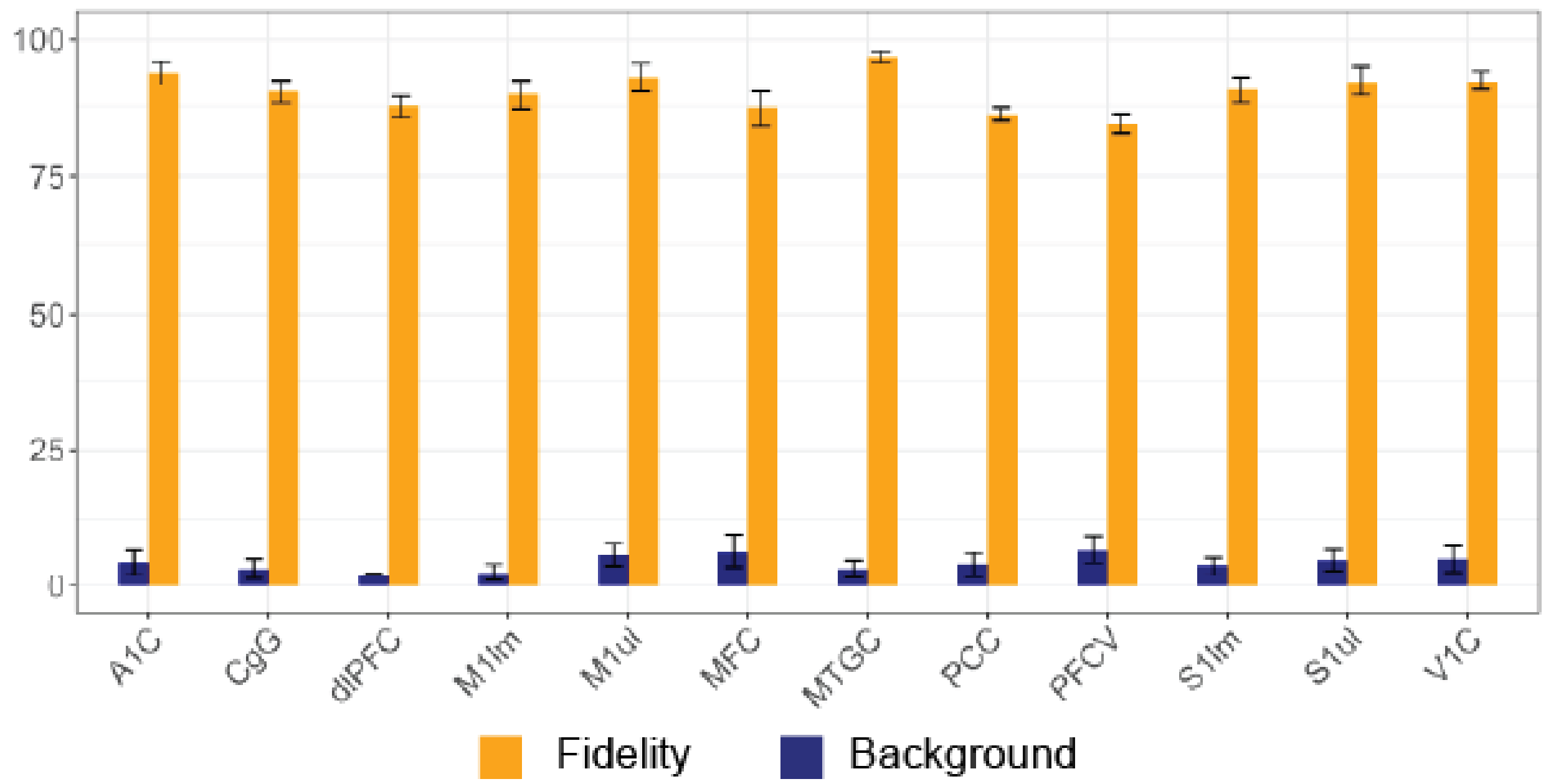

Supplementary Figure S10: High fidelity and low background levels of microglia subtype markers calculated using our method across all studies with subtype resolution.

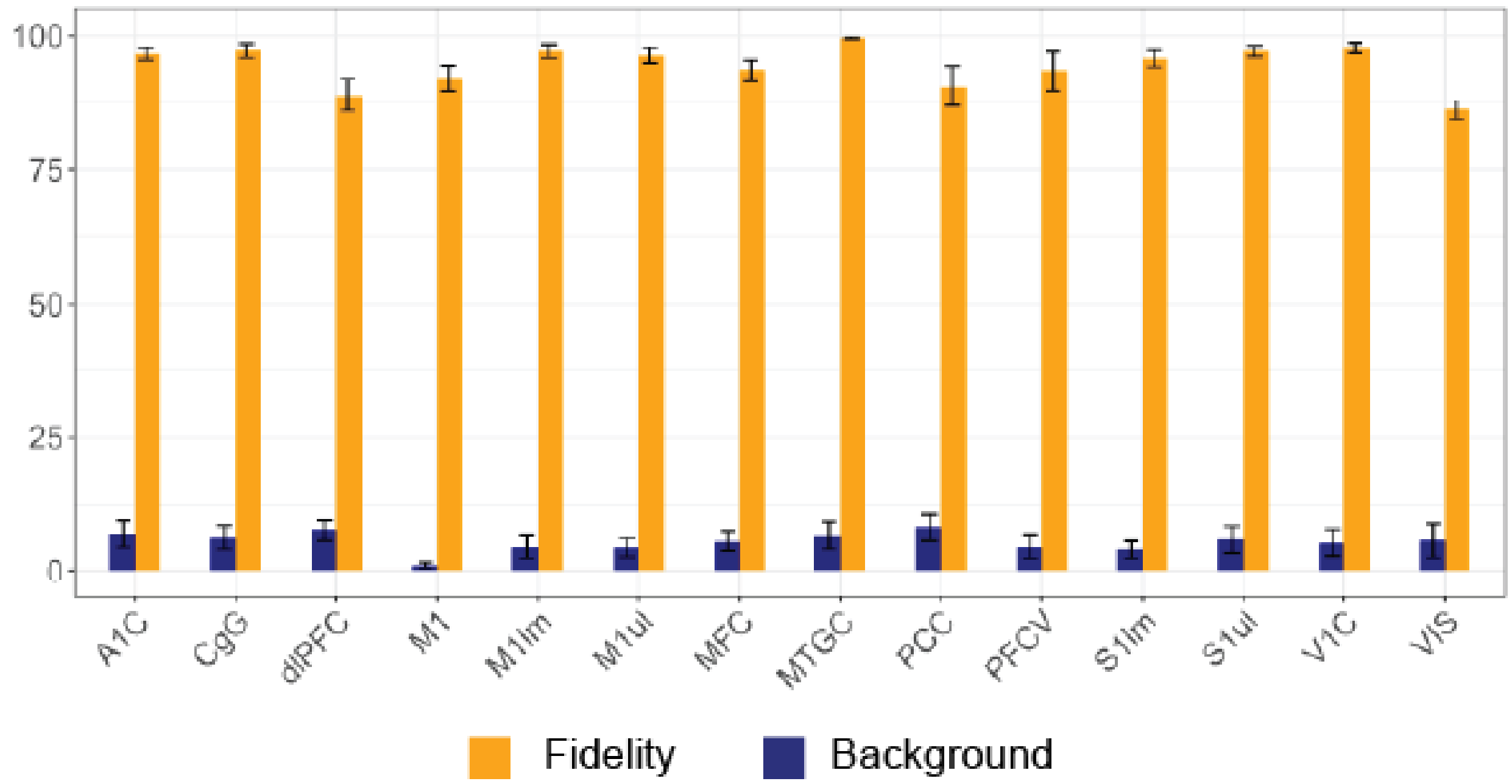

Supplementary Figure S11: High fidelity and low background levels of oligodendrocyte subtype markers calculated using our method across all studies with subtype resolution.

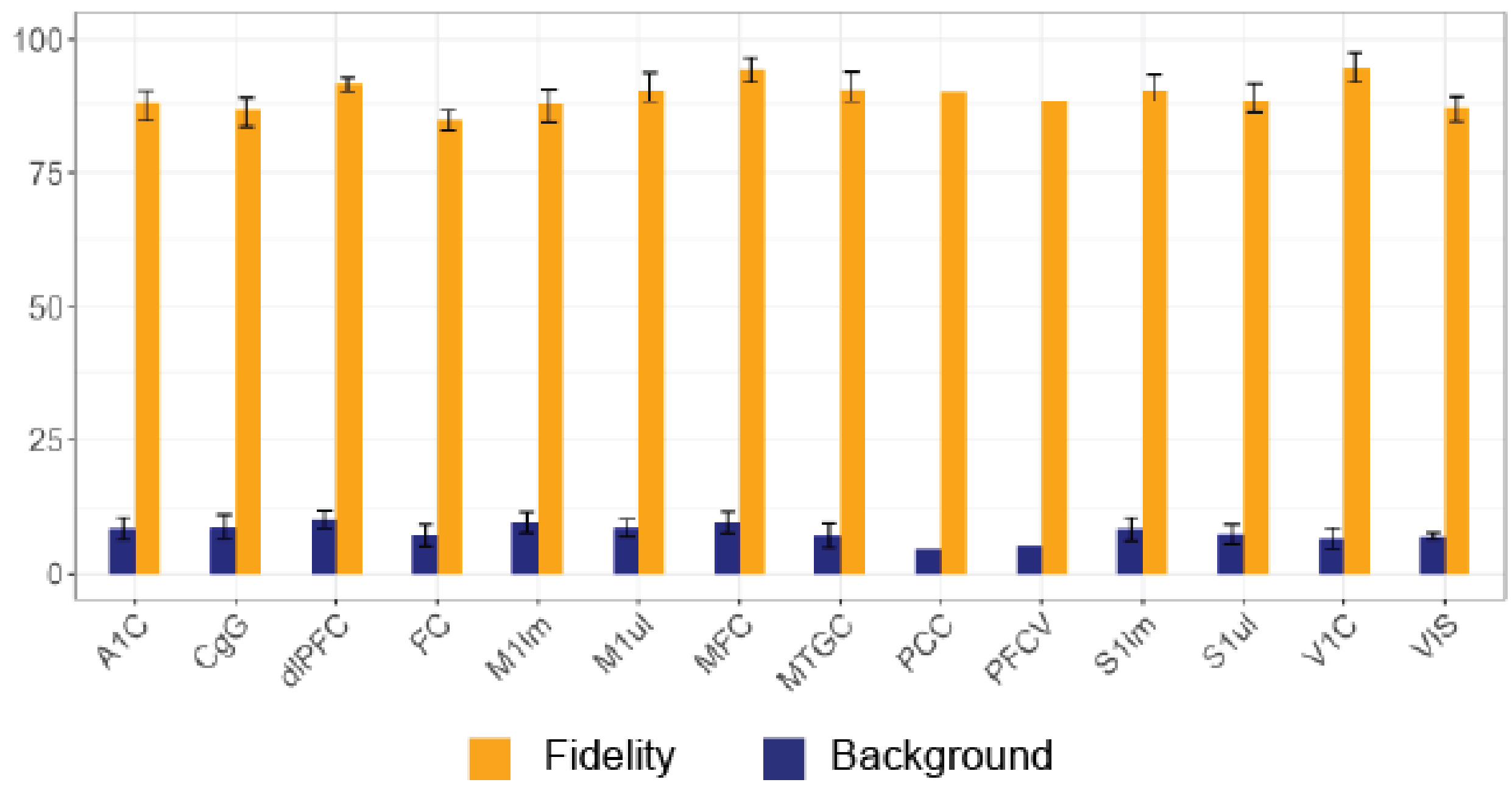

Supplementary Figure S12: High fidelity and low background levels of OPC subtype markers calculated using our method across all studies with subtype resolution.

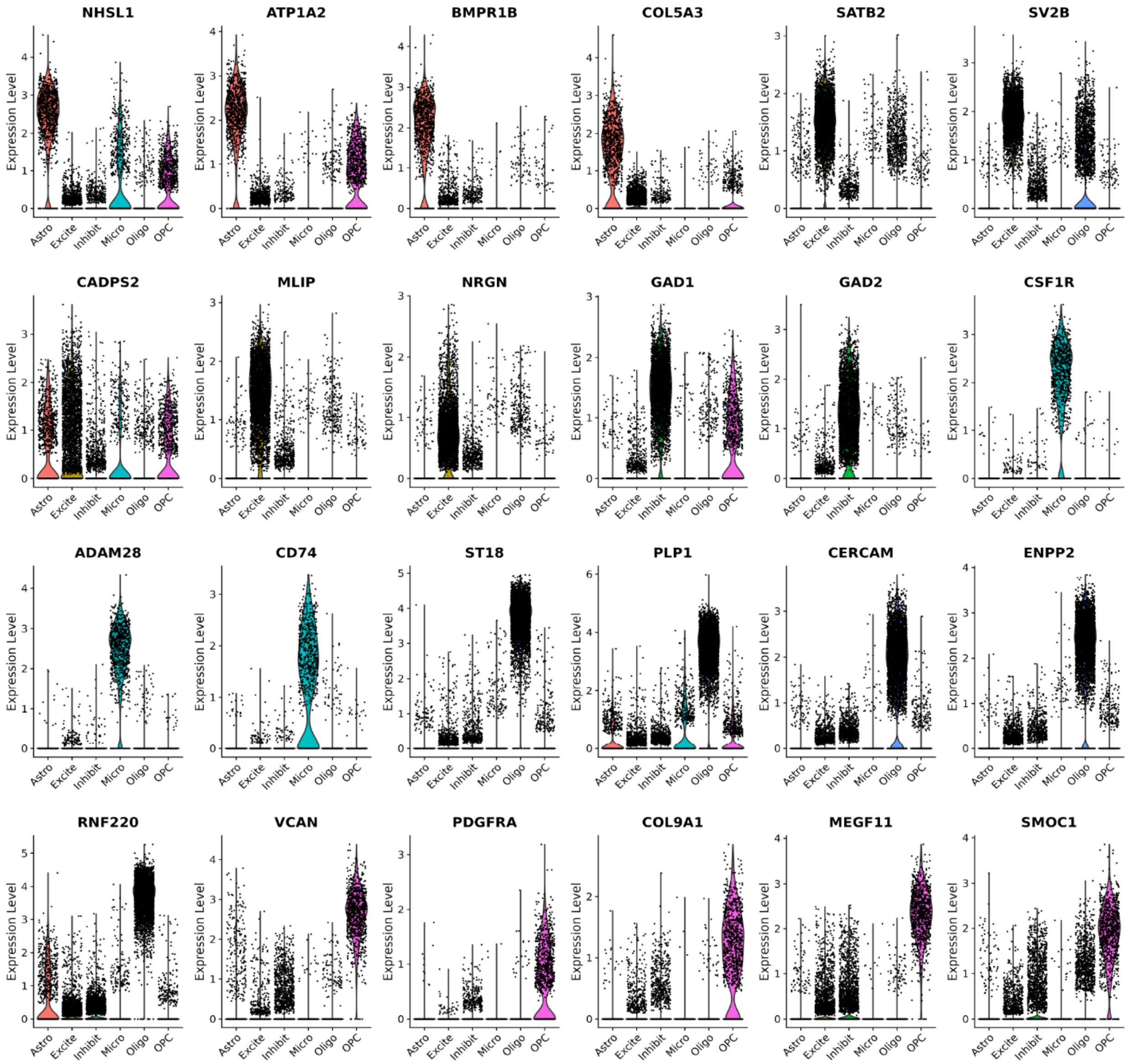

Supplementary Figure S13: Marker expression specificity of calculated markers in the hold out ACC dataset.

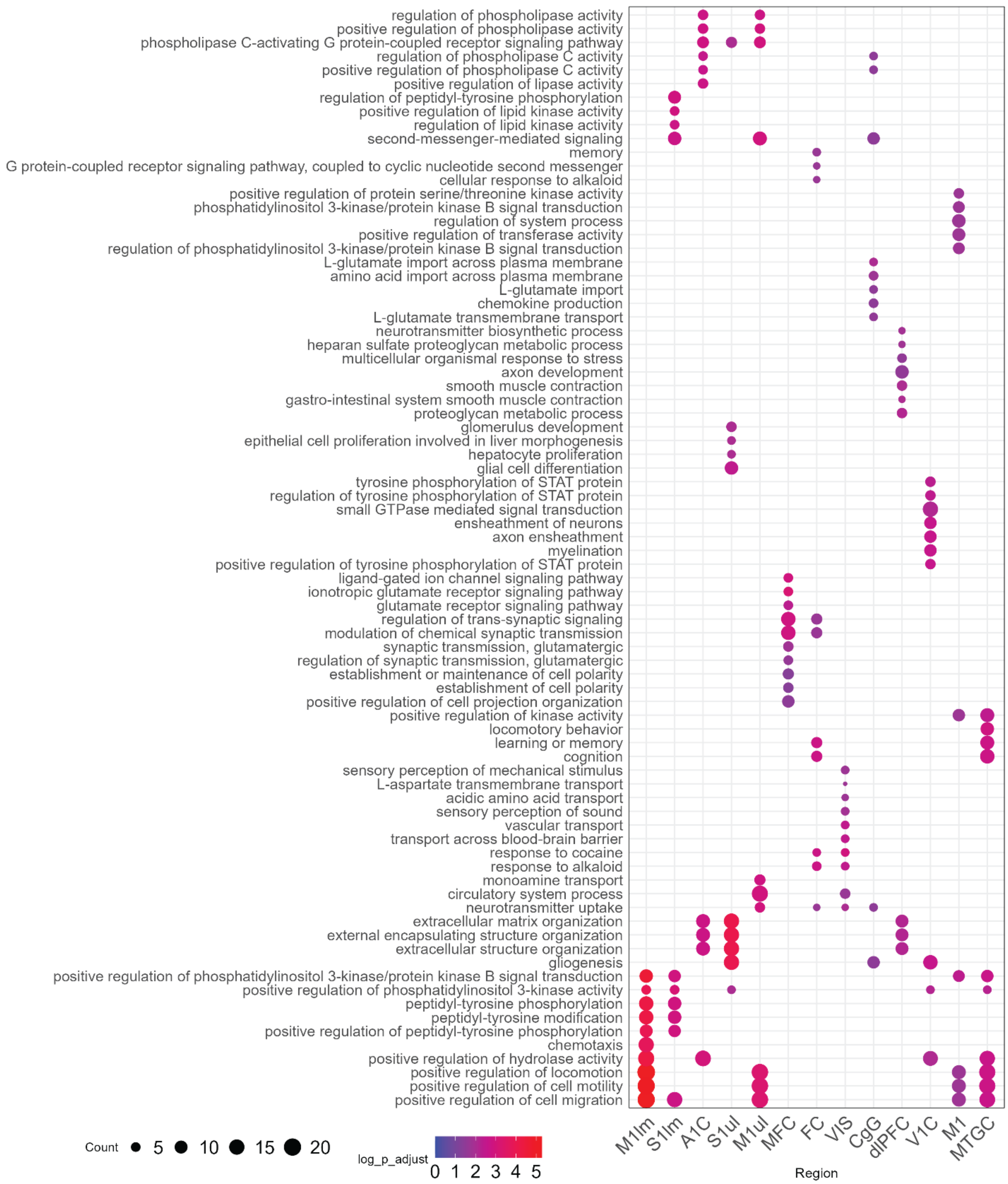

Supplementary Figure S14: Top 10 Gene Ontology Biological Process terms significantly enriched by subtype marker genes from different regions (p.adj value < 0.1).

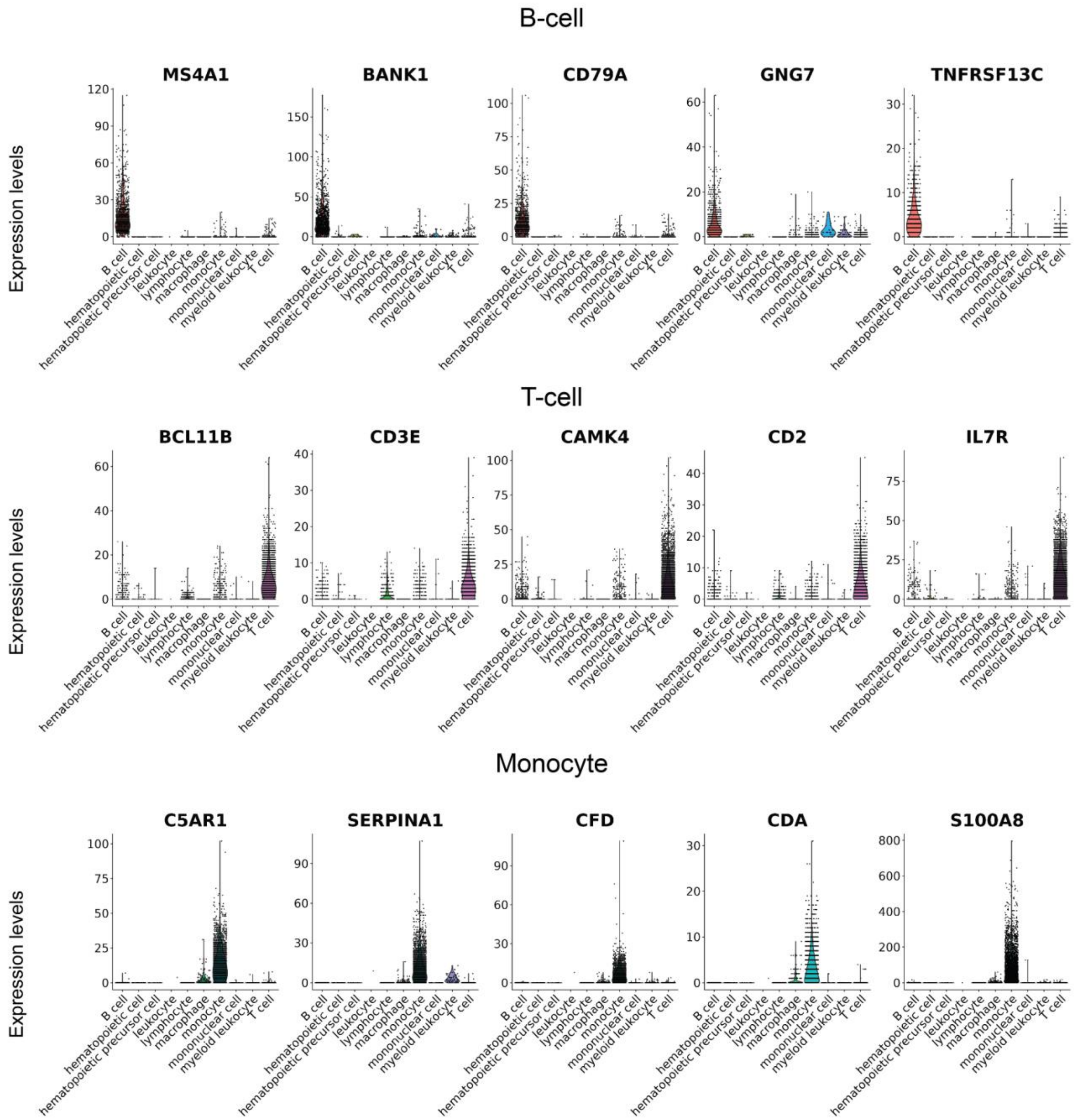

Supplementary Figure S15: Calculated PBMC markers derived from cell type specific fidelity and background thresholds for 3 major cell types in PBMC dataset – B-cell, T-cell, and monocytes.

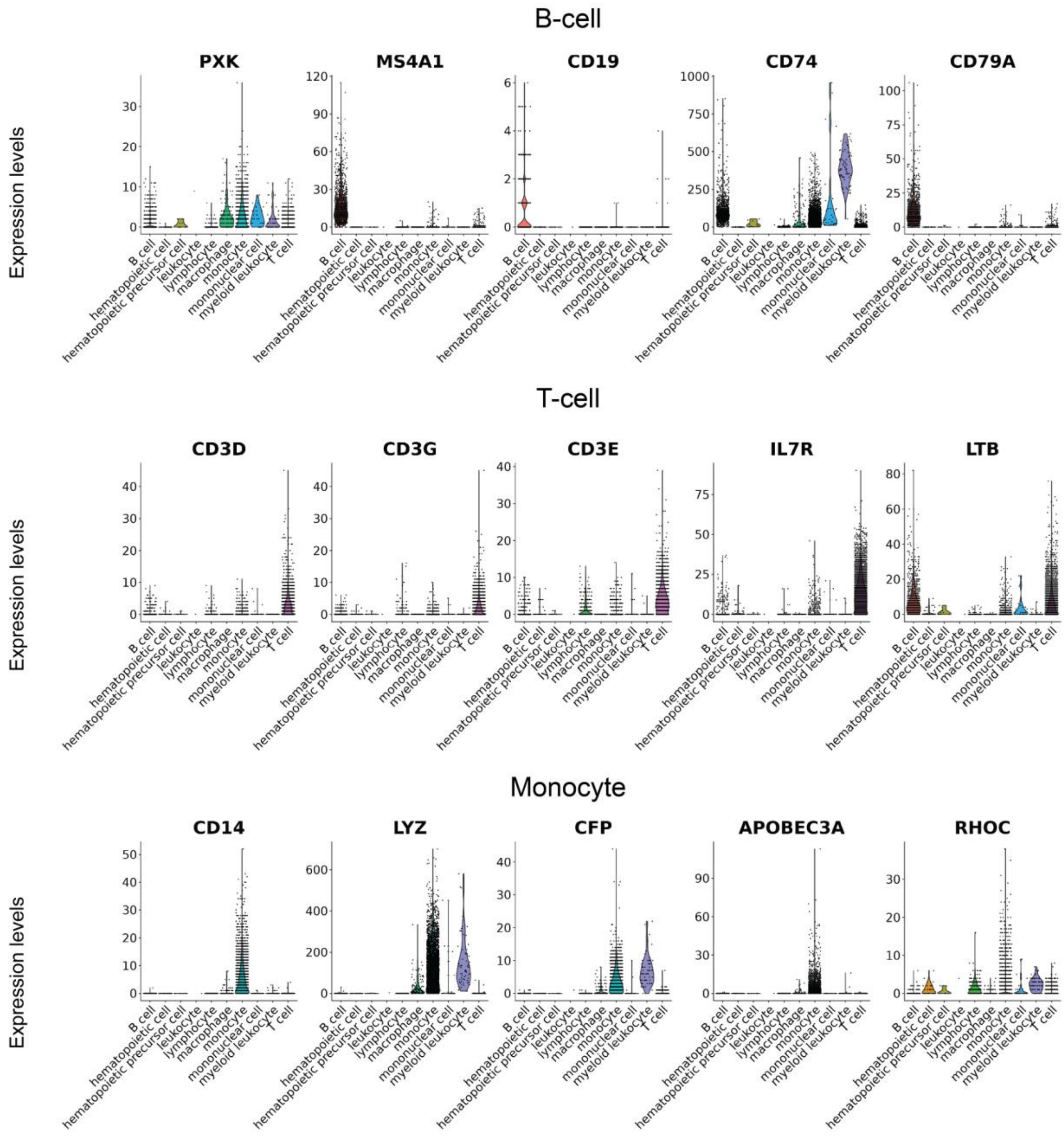

Supplementary Figure S16: Canonical PBMC markers derived from cell type specific fidelity and background thresholds for 3 major cell types in PBMC dataset – B-cell, T-cell, and monocytes.
